# An IoT-based healthcare system using blockchain technology and multiscale stacked Residual-GRU for secure data transmission

**DOI:** 10.1038/s41598-025-28856-z

**Published:** 2025-12-29

**Authors:** Anguraju Krishnan, Rajesh Arunachalam, S N V J Devi Kosuru, Swapna T, Sumanth Venugopal, Yogapriya J

**Affiliations:** 1 Department of Computer Science and Engineering, Saveetha School of Engineering, Saveetha Institute of Medical and Technical Sciences, Thandalam, Chennai, 602105 Tamil Nadu India; 2 Department of Electronics and Communication Engineering, Saveetha School of Engineering, Saveetha Institute of Medical and Technical Sciences, Thandalam, Chennai, 602105 Tamil Nadu India; 3 Department of Computer Science and Engineering, Koneru Lakshmaiah Education Foundation, Vaddeswaram, Guntur, 522502 Andhra Pradesh India; 4 Centre for Research Impact & Outcome, Chitkara University Institute of Engineering and Technology, Chitkara University, Rajpura, Punjab 140401 India; 5 Manipal Institute of Technology Bengaluru, Manipal Academy of Higher Education, Manipal, India; 6 Department of Computer Science and Engineering, Kongunadu College of Engineering and Technology, Tiruchirappalli, Tamil Nadu 621215 India

**Keywords:** IoT-based healthcare system, Medical data security, Multiobjective weighted restricted boltzmann machine, Magnified feeding-based american zebra optimization, Multiscale stacked Residual-Gated recurrent unit, Homomorphic polynomial encryption, Engineering, Mathematics and computing

## Abstract

This work resolves the crucial problem of secure data transmission in IoT-aided healthcare devices, where confidential patient data is vulnerable to breaches and cyberattacks. To resolve these complexities, this work proposes a novel secure data transmission system that combines blockchain technology, Multiobjective Weighted Restricted Boltzmann Machine (MW-RBM) for feature extraction, Magnified Feeding-based American Zebra Optimization (MFAZO) for weight optimization, and a Multiscale Stacked Residual-Gated Recurrent Unit (MSRes-GRU) for attack detection. The novelty of this work lies in combining residual GRU and blockchain for secure IoT healthcare data transmission, guaranteeing both transparency and attack detection. The improvement is displayed in weight optimization through MFAZO, which refines the feature extraction task and boosts the accuracy of the technique in attack detection. The designed approach involves gathering attack detection data, performing feature extraction utilizing MW-RBM with optimized weights and identifying IoT node attacks via the MSRes-GRU technique’s multiscale layers and the residual connections. The Homomorphic Polynomial Encryption (HPE) is further employed to secure the healthcare data during transmission. Lastly, the performance of the model is determined with conventional models. The accuracy of the designed MSRes-GRU is 96.22%, which is higher than the existing models such as DNN (85.65%), LSTM (80.71%), SVM (89.99%), and GRU (94.14%). The key results demonstrate the technique’s high detection accuracy and robust performance in recognizing the IoT-based attacks while guaranteeing effective, secure and transparent data transmission via blockchain. This research contributes to improving the secure and scalable IoT-enabled healthcare devices, providing a reliable model for trustworthy healthcare applications that preserve data integrity and privacy.

## Introduction

 The current technology revolutionized the healthcare industry. The efficiency and the quality of the healthcare industry improved suddenly because of technological development^1^. Both the patient and the doctor benefit from this technological advancement^2^. Compared with previous days, the present technology provides accurate and efficient lab reports, CT scans and MRIs. For patient care, the digital storage of medical records paves the way for utilizing Artificial Intelligence (AI) and deep learning^3^. Due to the advanced technology, it is possible to remotely monitor the patient through a technological device and gather real-time medical data from the patient using the IoT. This tends to perform medical diagnosis without any delay. For the secure transmission of medical details through the IoT network, there is a requirement for the encryption process^4^. The data encryption improves the security of the sensitive data and protects against attackers.

For data encryption, the cryptographic techniques are utilized, which ensure the integrity, authentication, non-repudiation and confidentiality^5^. The transmission of healthcare data tends to be more advantageous and allows the medical experts to diagnose the disease remotely and provide effective access to the specialist’s opinion, which saves cost and time. However, accessing and managing this medical data is not possible for the individual or the organization to handle^6^. Due to this, most organizations utilize the cloud storage platform for storing the medical data^7^. However, cloud service trustworthiness has gained more concern, which generally compromises data privacy^8^. Due to the sensitive nature of the medical data, storing the data directly in the cloud is more risky, and it affects the privacy of the patients^9^. The stored data in the cloud must ensure security to avoid vulnerability attacks on medical data. This enables addressing the integrity, availability and confidentiality^10^. Data encryption is the primary method to achieve confidentiality. The privacy-based concern in the IoT network tends to compromise the sensitivity, and passive and active attacks are performed on private data^11^. Sniffing the substantial data content from the public data is considered a passive attack. In an active attack, interference and alteration are performed to obtain access^12^. This tends to bad resource usage by the network user and complexity in the general communication flow^13^.

To manage the above-mentioned complexities, the integration of blockchain technology is necessary, which provides an effective solution in IoT-enabled healthcare systems. The digital information blocks in the blockchain include a chain of open databases^14^. It is not possible to modify the data, which is recorded in the immutable blockchain. The blockchain exhibits a decentralized system, other than immutability. In addition, the confidentiality between the parties is fostered by the smart contract in the data transmission process based on the self-executing and self-enforcing agreement terms^15^. In addition, the consensus procedure supported the blockchain for recording the distributed data integrity^16^. This resulted in trustworthy and secure patient medical data at the time of IoT-enabled transmission in the medical system^17^. An Intrusion Detection System (IDS) is considered the best option to mitigate attacks in the healthcare sector because it identifies the network’s abnormal behaviour^18^. In general, most of the existing IDS systems utilize the network data directly and perform poorly with the corresponding attack type regarding the false alarm rate and detection rate^19^. In addition, one of the significant challenges is scalability in IoT. More storage is required if there the more IoT devices, especially in the exponential data creation^20^. Therefore, this work implemented an effective approach for securing medical data transmission in an IoT-based framework based on the inclusion of the blockchain network.

**Novelty:** The novelty of this work lies in the seamless incorporation of distinct advanced techniques such as MW-RBM for feature extraction, MFAZO for weight optimization, MSRes-GRU for attack detection and HPE for secure data processing within a blockchain-aided healthcare IoT framework. While each component has ensured effective individual application, their integrated application resolves crucial complexities in healthcare data security and attack identification comprehensively. This integration allows accurate feature learning optimized for accuracy, effective detection of complex temporal attack patterns and privacy-preserving data encryption, all supported by the transparent, immutable, and decentralized nature of blockchain. Such a multi-layered and holistic mechanism is unprecedented in IoT healthcare devices, highly advancing effective, secure and trustworthy data transmission in this sensitive sector.

**Justification for choosing blockchain over other distributed Ledger technologies for healthcare:** The blockchain is chosen over other distributed ledger technologies for healthcare because of its special integration of decentralization, security, immutability and transparency, which are crucial for handling sensitive patient data. Unlike some distributed ledger technologies that prioritize the flexibility or speed at the cost of minimized security guarantees, consensus approaches of the blockchain, such as Practical Byzantine Fault Tolerance or Proof of Work, guarantee that once the data is recorded, it can’t be deleted or altered without the network consensus, thus safeguarding against unauthorized tampering modifications. This immutability is significant for maintaining trustworthy audit trails in regulatory contexts and healthcare compliance. In addition, the decentralized characteristic of the blockchain prevents the single points of failure common in conventional centralized models, improving the system’s resilience to attacks or outages. Its transparent ledger enables authorized stakeholders to independently validate the data integrity, fostering trust among regulators, providers and patients. While alternative distributed ledger technologies may provide merits such as quick transaction throughput, blockchain handles the balanced trade-off among transparency, security and decentralization, making it well-suited for the privacy-sensitive, high-stakes environment of IoT-aided healthcare devices.

**Contributions:** This article makes the following contributions to the field.

- To design a novel attack detection and IoT-enabled secure blockchain framework for effective data transformation in healthcare systems using deep learning and cryptography methods. This framework provides an efficient and secure mechanism to transmit healthcare data between devices.
- To perform an optimal feature extraction in medical data, the MW-RBM is constructed, in which the weight is optimized using MFAZO. This algorithm is designed by modifying the traditional AZOA to improve the feature retrieval.
- To implement an MSRes-GRU technique for accurate attack detection, which is built by the combination of the residual GRU with the added operation of a multiscale stacked mechanism. This approach effectively detects the attack in the IoT medical nodes based on the given attack detection dataset and generates an accurate result.
- To implement an encryption mechanism in the blockchain network named HPE that improves the security of the healthcare data by ensuring it is protected from vulnerable attacks. This mechanism supports various security protocols in the medical field and allows secure communication between multiple parties.

**Fig. 1:**
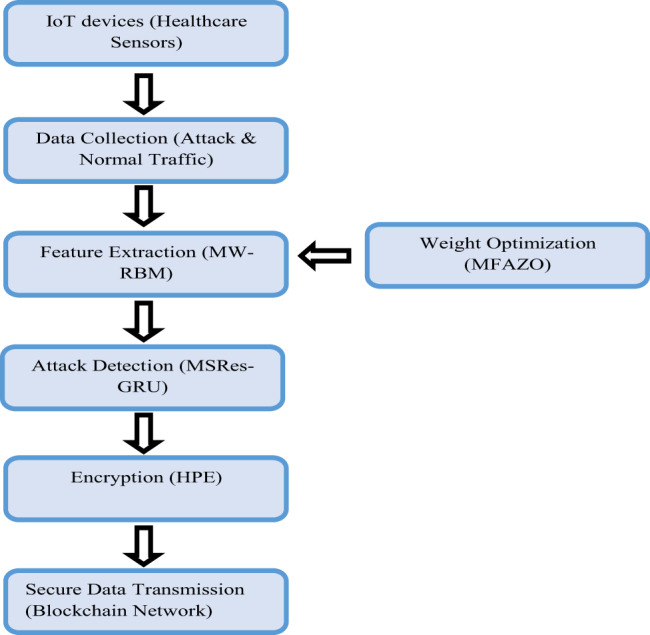
Flow diagram of the designed model

**Flow of the proposed work**:

The suggested work introduces a unified model that strategically combines the residual GRU, blockchain and weight optimization to improve the data transmission in IoT-aided healthcare devices securely. The operation starts with attack-based data garnered from IoT systems, which is further passed via the MW-RBM for feature extraction. To enhance the relevance and quality of the extracted features, the MFAZO is employed, optimizing the feature weights based on the multi-objective parameters. These optimized features are further subjected to the MSRes-GRU technique, which employs the residual connections and multiscale layers to efficiently learn the temporal patterns and identify the malicious activity in IoT nodes. Once estimated, the healthcare data is encrypted employing HPE and sent via a blockchain network, guaranteeing transparency, integrity and tamper-proof logging. This seamless integration of approaches enables the model to perform threat detection, secure data management and effective transmission in a robust and unified way.(Fig. 1)

**Organization:** The rest of the article is organized as follows. Section 2 discusses the traditional approaches related to this framework. Section 3 presented the proposed smart healthcare system in detail with the optimization mechanism. Section 4 explained the optimal feature selection and its objectives. Section 5 demonstrates the detection model and the encryption mechanism. Section 6 presents the research outcomes. Section 7 concludes the work with future directions.

## Literature survey

### Related works

In 2023, Kaur et al.^21^ have implemented a secure and efficient approach for the IoT network. The network security was improved by involving the matrix XOR with the cryptography model. The zoning evolution was employed to optimize the functionality along with the adaptive mutation process. This was achieved by fine-tuning the hyperparameters to enhance the overall efficiency. The low complexity cryptography was used to encrypt the confidential data from the IoT. The image cover block was used to embed the encrypted data based on the selected algorithm. The communication of the IoT framework was ensured by this process, and it could be easily decrypted by the user end. The obtained results were superior to other competitive approaches.

In 2022, Randhir et al.^22^ have suggested an integrated framework that included the smart contract in the blockchain technology along with the deep learning approaches. The novel data-sharing framework was designed to authenticate, verify and validate the communication transaction within the network. Only the authenticated data based on the consensus mechanism in blockchain could be determined using the deep learning approach. Here, the hybrid integration scheme was developed to encode the healthcare data and determine the attack detection process. The superiority of the scheme was ensured by the experimental results in the security analysis process.

In 2023, Rashid et al.^23^ have designed a distributed sensing model for ensuring the security of the collected medical data. Blockchain mechanism and the encryption algorithm were used to prevent the data from other harmful attacks. Several datasets were leveraged to validate the efficacy of the recommended scheme along with various metrics like scalability, throughput, reliability and security. The effectiveness of the architecture was highlighted by the obtained result. In addition to the AUC analysis, there was a significant improvement in the implemented model.

In 2023, Taloba et al.^24^ have adopted the IoT in the healthcare sector to improve patient care based on initiating the distribution of medical resources. Distinct concerns were caused in IoT systems. To tackle the challenges, one of the effective ways was to introduce blockchain technology for managing the security of the real-time control system. From the results, blockchain mechanism provided a secure approach for the healthcare field by generating a hash of each data point to avoid threats.

In 2023, Kumar et al.^25^ have suggested a new mechanism for developing a scalable blockchain framework to enhance security and integrity. The off-chain storage system was integrated with the deep learning based blockchain framework for secure transmission of healthcare data. This solves the wastage of the data storage cost using the Ethereum smart contract. The deep learning architecture was designed using the authenticated data to identify the intrusion in it. Later, the combinational model was designed for detecting the intrusion, and the output showed a higher accuracy for the presented datasets.

In 2024, Kanwal et al.^26^ have introduced a novel encryption scheme based on IoT blockchain to ensure the authenticity and integrity of the medical data. Some of the performance measures, like the key sensitivity analysis, histogram, key space analysis, etc., were used to examine the suggested model. The outcome from the results demonstrated that the implemented model was highly effective in preventing security concerns and improving data integrity.

In 2024, Rashid et al.^27^ have implemented the optimization algorithm and the standard scheme for the effective transmission of healthcare data using the key encryption model. The developed model converted the plain text into ciphertext based on the secret key generation. This ensured the security against manipulation and unauthorized access. The suggested framework was validated by developing the attack scenario in the IoT-based collected data. The security algorithm was compared with the proposed system under the simulation setup. The result highlighted that the designed system has a better accuracy value than other traditional security models.

In 2023, Bhan et al.^28^ have designed the blockchain-based Hyperledger technique for the secure transmission of medical data that was fetched using the IoMT. The patient’s confidential records were protected by suggesting the clustering management system, which helped to stop the malicious nodes. Additionally, the cryptography model was included to protect the medical data against vulnerable attacks. In the end, the simulation outcomes explained that integrating the blockchain into the detection performance resulted in a more effective scheme than the other available schemes in the medical industry. High reliability and security were attained from the results, showing the efficacy of the suggested technique.

In 2025, Ullah et al.^29^ have suggested a new deep network for performing personalized diagnosis and treatment procedures. This model enhanced the interpretability via XAI models and improved the privacy through federated learning.

In 2024, Adil et al.^30^ have provided a detailed survey by concentrating on the AI, machine and deep learning-enabled models. These models were validated based on their security and their merits and demerits were listed. Finally, the open security challenges were discussed.

In 2025, Sharma and Shambharkar^31^ have reommended a model by integrating deep learning and blockchain for the diagnostics and management of medical data. This approach improved the data security and also displayed superiority over other approaches. In 2025, Sharma and Shambharkar^32^ have introduced a dynamic adaptive DRL model for addressing the real-time threats. This model outperformed the conventional encryption approaches and also guaranteed real-time threat mitigation and improved data integrity.

In 2025, Sharma and Shambharkar^33^ have suggested a hybrid security model by including blockchain and deep learning for advanced threat detection. This model underscored the efficacy of the designed system in improving scalability and security.

### Research gaps and challenges

Secure data transmission in the healthcare industry uses IoT technology for data transmission, which is important to seek medical assistance for patients in remote regions. The data transmission is prone to many challenges, such as scalability limitations with large data, privacy concerns related to healthcare data transmission, data vulnerability in blockchain and complexities in integrating this complex system with existing healthcare infrastructure. These issues need to be addressed to ensure real-time reactiveness for critical data transmission. Table 1 offers the features and complexities of traditional healthcare data transmission models. The following section lists the research challenges determined from the previous models, which will be addressed in the proposed work.

- The conventional approaches fail to address the complex temporal dependencies in IoT data because of the basic recurrent models or shallow architectures, resulting in poor attack detection accuracy.
- Mostly, the conventional approaches lack integration with secure models such as blockchain, making them susceptible to data breaches and tampering.
- The conventional feature extraction approaches struggle to manage the high-dimensional data and the multi-objective parameters, minimizing their effectiveness in recognizing the subtle attack patterns.
- The majority of the approaches employ static or manually tuned weights, which fail to adapt to dynamic data patterns, leading to suboptimal performance.
- The previous approaches employing conventional encryption approaches lack support for executing the encrypted data, limiting secure computation capabilities.

The proposed integration of residual GRU and blockchain resolves the problems of conventional IoT-based healthcare security approaches by combining deep temporal learning with decentralized, tamper-proof data management. The residual GRU model captures long-term and complex temporal patterns in IoT data via gated approaches and residual connections, allowing relatively accurate and real-time attack detection, even in large-scale environments. Also, the utilization of blockchain guarantees that all transmitted data is securely time-stamped, hashed, and stored in an immutable ledger, resolving the lack of trust and transparency in previous approaches. This synergy not only improves the system against cyberattacks but also offers an auditable, scalable, and secure model for sensitive healthcare data transmission, something the traditional approaches fail to attain because of the weak or isolated security approaches.

**Table 1:** Features and challenges of data transmission model in IoT-Enabled healthcare Systems.

Author [citation]	Methodology	Features	Challenges
Kaur et al. [18]	LCEGC	The model provided effective data encryption in less time, compared to traditional methods.	The optimization algorithm is not well explored for further development of the model.
Randhir et al. [19]	PBDL	It offers high scalability to access healthcare data.	The system requires better performance in a defined software network.
Rashid et al. [20]	SSCA	Important concerns such as response time, scalability and security have better performance than conventional methods.	It needs further clarification in handling enormous data while ensuring data protection.
Taloba et al. [21]	Blockchain-based Hybrid Model	This system performs effective detection of malicious attacks and illegal behaviours.	This model is complex and costly.
Kumar et al. [22]	BDSDT	The model is secure for data transmission and usage.	This system requires advanced adaptability and scalability for improved performance.
Kanwal et al. [23]	CTMES	The developed model is versatile and can be applied to wide applications.	The integration of systems with advanced technology needs further development.
Rashid et al. [24]	WbAES	This system is well-versed in detecting security threats and providing data privacy.	There is no accurate and automated system for performance.
Bhan et al. [25]	HSN	It is a robust and lightweight model compared to conventional methods.	Advancement for hiding node identity to preserve patient details is required.

## Illustration of an effective model of a smart healthcare system with data collection and algorithm

### The developed smart healthcare system and its process

**Fig. 2:**
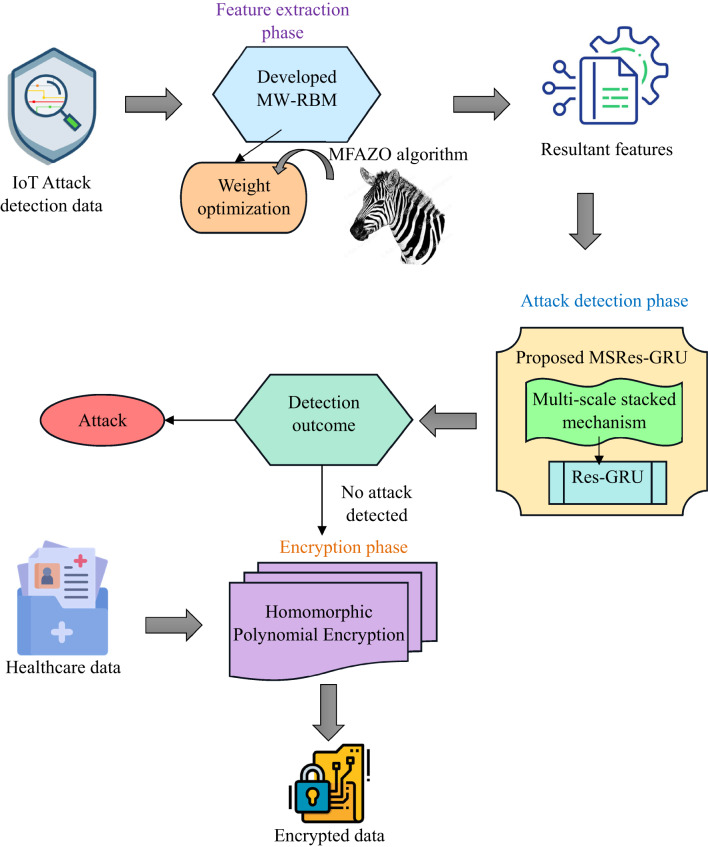
Structural diagram of the implemented IoT-Blockchain-enabled healthcare management system using deep learning.

In the present time, one of the fastest-developing technologies in the computing field is IoT, where the connectivity among different types of devices is unified. Various fields utilize IoT because of its adaptability, energy efficiency and portability. In the healthcare domain, the IoT plays a major role, solving the challenges in standard healthcare facilities. IoT in healthcare systems helps to monitor the real-time activity of humans, which keeps track of the patient record. From the healthcare management huge amount of data is garnered by the sensor. Preserving the security of this huge data is more complex. Also, some other challenges like availability requirements, data storage and reliability happened. In general, the client database is used to save the electronic-based medical reports, where the coordinator from central has access control to delete or update the medical data. This tends to be data loss or data tampering. Hence, there is a requirement for the distributed network to access the medical records with enhanced security. Distinct deep learning and machine learning approaches were developed in the earlier literature. However, there is still a security issue in storing medical data, due to its highly sensitive nature. Therefore, this work developed an effective model to ensure that medical data is protected from unauthorized access using blockchain technology and the deep learning approach, and its working flow is displayed in Fig. 2.

The major goal of this framework is to develop an advanced methodology for the IoT-enabled healthcare system to ensure security in the transmission of healthcare data. This work is constructed with four stages, such as data collection, optimal feature extraction, attack detection and encryption for secure medical data. At first, the available attack detection dataset and medical dataset are taken from an online resource. Further, the optimal feature selection is carried out to perform effective feature extraction from the given attack detection data. Here, the developed MW-RBM is utilized, which is constructed by implementing the RBM model with the weight optimization mechanism to achieve an accurate result. The RBM feature is extracted, where the weight in the model is optimized by implementing the MFAZO algorithm. The implemented MFAZO is constructed by refining the random integer in the existing AZO algorithm to improve the optimal result. Further, the extracted feature is used to detect the attack in the IoT node in the detection stage. Here, the attack detection is performed utilizing the MSRes-GRU model for an effective result. Here, the MSRes-GRU model is developed by the combination of the residual network with the GRU, along with the multi-scale stacked concept. Once the attack is detected, the healthcare data requires a secure storage and transmission system to avoid leakage of sensitive data. Therefore, the HPE-based encryption is carried out in the blockchain platform to encrypt and decrypt the healthcare data for security. Finally, the comparative analysis is performed to determine the efficiency of the implemented model. From the results, the developed model ensures the security of the healthcare data without any vulnerability attacks.

### Collection of healthcare data

For the proposed data transmission in an IoT-based healthcare system, the diabetes dataset is utilized. The dataset is taken from an online available resource and its detail is given below.

**Attack Detection dataset**: The corresponding dataset is accessed from the link “https://www.kaggle.com/datasets/venkatakanumuru/kddcup99csv: Access date: 2025-02−14”. The dataset is mainly used to train the deep learning approach to identify attacks in the IoT node. The data source includes the details of some attacks in the IoT healthcare system.

From the data source, the collected attack data are specified as $A{d_k}$. Here $k=1,2,3, \ldots ,K$. The total number of collected data is termed as $K$.

**Healthcare dataset**: The diabetes dataset employed in this work is taken from the link “https://www.kaggle.com/datasets/akshaydattatraykhare/diabetes-dataset Access date:2025-02−06”. The dataset holds the information of digestive, diabetes and kidney diseases. The dataset is mainly developed to forecast whether the patient has diabetes or not based on the corresponding measures used in the dataset.

From the obtained dataset, the garnered diabetes data are indicated as $D{b_w}$. Here $w=1,2,3, \ldots ,W$. The total number of collected data is termed as $W$.

### Blockchain novel Meta-Heuristic algorithm: MFAZO

In this framework, the MFAZO algorithm is designed to incorporate an effective feature extraction process to select the optimal feature set.

**Purpose:** In the implemented attack detection model, the MFAZO algorithm is designed to perform optimal feature selection. The weight in the MW-RBM model is relatively significant to improve the performance of the feature selection and to address the training complexity. Hence, the MFAZO algorithm is developed in this article, which optimizes the value of the weight parameter in the MW-RBM approach. This optimal feature selection process improves the functionality of the approach by determining the highly minimizes the training time for further processing.

**Novelty:** The designed MFAZO is the updated version of the traditional AZOA^34^. The baseline AZOA is influenced by the social behavior of the American zebra, especially the leadership exercise. The AZOA can generate the optimal solution by managing the exploitation and the exploration balance. This supports to tackle various real-world engineering issues. Compared with the other metaheuristic algorithms, the AZOA is more efficient and has a smaller width in the optimization process. In addition, this algorithm provides the finest feasible solution and generates better convergence. However, while determining the baseline AZOA, the feeding activity in the exploration phase leverages a random factor in the interval of 0 to 1. The iteration gets hindered by this factor and results in an undefined solution for the complex problem. Hence, this random value in the AZOA is updated and designed the MFAZO algorithm. The updated random value $R3$ is formulated by Eq. (1)

1 $$R3=t \times \frac{{0.02}}{{Ma{x_{itr}}}}$$

Here, the term $Ma{x_{itr}}$indicates the maximum iteration, and the modified random value is represented as $R3$. This value has been updated in the feeding activity of the baseline AZOA, and it is mathematically derived in Eq. (2).

2 $$\bar {W}_{i}^{j}=\left\{ {\begin{array}{*{20}{c}} {2R1\,\sin (2\Pi {R_2}) \times (W_{u}^{j} - W_{i}^{j})+W_{u}^{j}}&{R3<0.5} \\ {2R1\,\sin (2\Pi {R_2}) \times (W_{u}^{j} - W_{i}^{j})+W}&{Otherwise} \end{array}} \right.$$

Here, the ${i^{th}}$zebra stallion position is indicated as $W_{u}^{j}$and $W_{i}^{j}$. The random value $R1$is selected within the range of$[ - 2 - 2]$. The adaptive parameter is specified as $R2$and $R3$ is the random value that is updated by Eq. (1).

**Theoretical justification of MFAZO’s modification:** The designed MFAZO improves upon the conventional AZOA by refining the random value generation approach to improve the balance among exploration and exploitation during the search task. This modification enables the designed MFAZO to adaptively handle the diversity of the candidate solutions, eliminating the premature convergence to local optima, an ordinary issue in AZOA. By magnifying the feeding behavior simulation dynamically, the MFAZO enhances the ability of the algorithm to discover the large search space early on, while gradually concentrating on promising areas in subsequent iterations. The empirical outcomes illustrate that this results in faster convergence and more reliable recognition of near-optimal or optimal solutions, contrasted to AZOA, making MFAZO a highly robust and effective optimizer for complex multi-objective issues such as weight optimization in IoT healthcare data 

**Algorithm 1:**
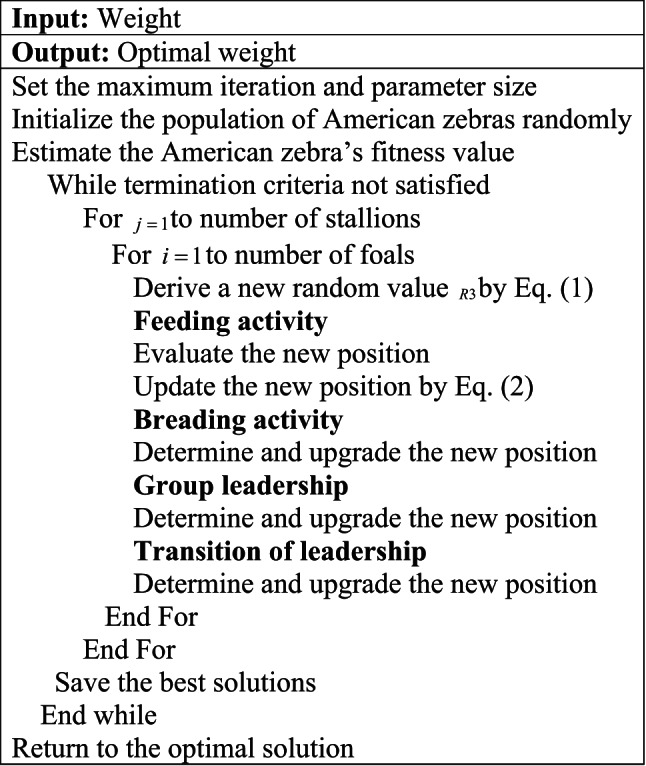
Suggested MFAZO

## Optimal-based feature selection scheme and its objective function for healthcare system

### Restricted Boltzmann machine

RBM^35^ is the type of undirected graphical model that has the observed random variable and the hidden layer. The hidden unit is considered to be one of the significant feature extraction parts in the RBM structure, which is based on the observed data. For each feature, the corresponding hidden unit is independent and makes the RBM more valuable. By using the Gibbs distribution, the observed variables are evaluated using Eq. (3).

3 $$r(v;\theta )=\frac{1}{{L(\theta )}}\sum\limits_{g} {\exp ( - E(v,h;\theta ))}$$

Here, the set of parameters is considered as $\theta$. The hidden variable and the observed variable are represented as $h$ and $v$. The generalized constant $L(\theta )$is formulated using Eq. (4).

4 $$L(\theta )=\sum\limits_{u} {\sum\limits_{g} {\exp ( - E(v,h,\theta ))} }$$

The free energy in the RBM parameter is specified as $D(v,g,\theta )$ and computed using Eq. (5).

5 $$E(v,h,\theta )= - \sum\limits_{{j,l}} {{v_j}{y_{j,l}}{h_l} - \sum\limits_{j} {{f_j}v{}_{j} - } } \sum\limits_{j} {{g_l}h{}_{l}}$$

Here, the terms $j$ and $l$ denote the sequence member and $\theta$is set of node parameter. The variable distribution begins when there are no balanced hidden variables, and it is determined using Eqs. (6) and (7).

6 $$q({g_l}|v)=\sigma ({f_l}+\sum\limits_{j} {{v_j}{x_{jl}})}$$

7 $$q({v_l}|g)=\sigma ({b_j}+\sum\limits_{l} {{x_{jl}}{g_l})}$$

Here, the variable$\sigma$ is the logistic sigmoid function. The obtained result extracts the features from the given data with the help of the distribution network.

### Proposed MW-RBM

The significant feature extraction is considered to be one of the major challenges in the detection phase. There are several feature extraction techniques are there in the existing works. However, the existing techniques haven’t been able to reach higher accuracy results in the feature extraction process. Therefore, the MW-RBM-aided feature extraction is presented in this work.

**Difference between MW-RBM and standard RBM:** The designed MW-RBM varies from the conventional RBM by including the multi-objective optimization and adaptive weighting strategies tailored for the complex nature of the IoT healthcare attack identification. While the existing RBM learns from feature representations by designing the input data’s joint probability distribution in an unsupervised way, the designed MW-RBM improves this by allocating dynamic weights to distinct features according to their relevance to distinct objectives, such as minimizing the false positives and maximizing the detection accuracy. This weighted mechanism enables the designed MW-RBM to concentrate more on crucial features that represent the powerful attacks while suppressing noisy or less informative data common in heterogeneous IoT healthcare environments. In addition, the multi-objective model allows the framework to balance the competing objectives, such as computational efficiency and precision during training, resulting in highly discriminative and robust feature extraction. Thus, the designed MW-RBM provides a highly effective and fine-tuned representation learning task than existing RBMs, improving the capability to identify diverse and subtle attack patterns in IoT healthcare devices.

Here, the gathered attack detection data$A{d_k}$ are provided as input to the feature extraction phase. For extracting related features, the high-level feature and the low-level feature are integrated into the RBM model. Even though the RBM feature extraction performs better, it also has the limitation of less generalization capability. In addition, it has a specified training rate, which is non-conductive. Due to these limitations, the RBM faces complexity in extracting the corresponding information. Therefore, based on the training sample, the parameter adjustment is significant in the training phase to achieve an effective result. The nonlinear function configured by the nonlinear sigmoid block generally depends on the weight. This weight optimization is required to improve the feature extraction process. In this, the weight is optimized utilizing the suggested MFAZO algorithm to effectively improve the feature extraction from RBM. The objective function is formulated by Eq. (8).

8 $$OF=\mathop {\arg \,\,\hbox{max} }\limits_{{\left\{ {W{t^{uo}}} \right\}}} \left[ {Rs+Cs+Cc} \right]$$

Here, the term $W{t^{up}}$is the optimized weight, which ranges between [−20, 20]. The optimized weight is further calculated along with the already trained weight from the RBM technique to generate the new weight value as given in Eq. (9).

9 $$Wt=wt+wt \times \frac{{W{t^{up}}}}{{100}}$$

Here, the term $wt$is the already trained weight in the RBM model. The final weight to generate the result is termed as $Wt$. The process of this optimization helps in achieving maximum relief score$Rs$, chi-squared statistic$Cs$ and correlation coefficient$Cc$in the optimal feature selection process.

The relief score determines the relevance of the feature that discriminates among various classes in the data source. It also computes how well the feature determined the instances of the same and the distinct classes, and it is defined in Eq. (10)

10 $$\operatorname{Re} f={x_{_{i}}} - ({y_i} - S{t_i})+({y_i} - D{t_i})$$

Here, the instances of the same and the distinct classes are indicated as $S{t_i}$and $D{t_i}$.

The chi-squared statistic is the hypothesis test that is used in determining whether the variables in the test statistics are independently influencing.

11 $$Cs=\sum {\frac{{{{\left( {xv - yt} \right)}^2}}}{{yt}}}$$

Here, the terms $xv$and $yt$indicate the observed and the expected values.

The correlation coefficient is considered to be the numerical measure that determines the statistical relation between the two variables. It helps to calculate the strength of the variables’ linear relationship and is evaluated using Eq. (12).

12 $$Cc=\frac{{\sum {(k - \bar {k})(j - \bar {j})} }}{{\sqrt {\sum {{{(k - \bar {k})}^2}\sum {{{(j - \bar {j})}^2}} } } }}$$

Here, the terms $xv$ and$yt$ are the variable and its mean value is indicated as $\bar {j}$and$\bar {k}$. With the help of the implemented RBM and the multi-objective weight optimization, the accurate feature is extracted, and it is represented as$Ad_{k}^{{fe}}$.

**Weight optimization process:** The weight optimization task in this work employs the MFAZO, an evolutionary algorithm motivated by the American zebra’s natural foraging behavior. The MFAZO iteratively searches for the optimal weights to improve the MW-RBM’s feature extraction capability. It balances distinct objectives like maximizing the detection accuracy while minimizing the computational burden by adjusting the weights dynamically on the basis of candidate solutions’ fitness validations. Through approaches such as exploration and exploitation, the designed MFAZO effectively navigates the search space to prevent the local optima, guaranteeing that the MW-RBM retrieves the highly related discriminative and related features from IoT healthcare data. This optimized weighting highly improves the performance of the subsequent attack detection by the residual GRU approach, resulting in highly accurate and reliable detection of security threats.

**Weight optimization process**: The weight optimization task in this work employs the MFAZO, an evolutionary algorithm motivated by the American zebra’s natural foraging behavior. The MFAZO iteratively searches for the optimal weights to improve the MW-RBM’s feature extraction capability. It balances distinct objectives like maximizing the detection accuracy while minimizing the computational burden by adjusting the weights dynamically on the basis of candidate solutions’ fitness validations. Through approaches such as exploration and exploitation, the designed MFAZO effectively navigates the search space to prevent the local optima, guaranteeing that the MW-RBM retrieves the highly related discriminative and related features from IoT healthcare data. This optimized weighting highly improves the performance of the subsequent attack detection by the residual GRU approach, resulting in highly accurate and reliable detection of security threats.

**Fig. 3:**
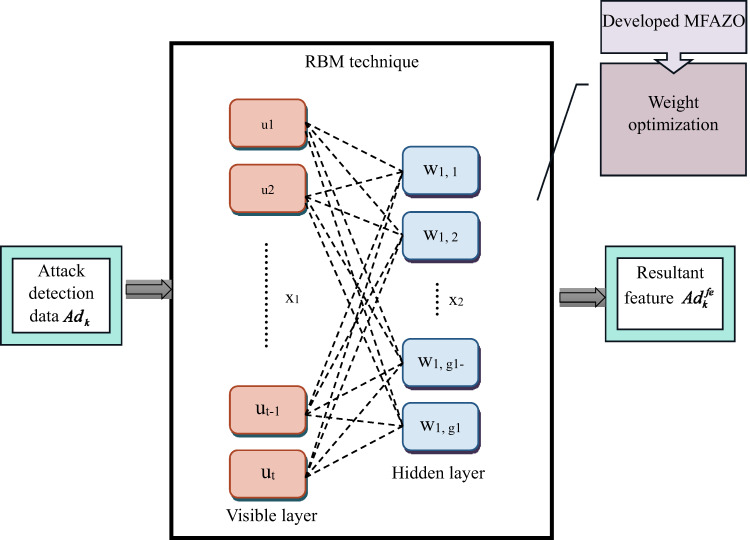
Structural diagram of MW-RBM for feature extraction.

**MFAZO’s balance of multiple objectives during weight optimization:** The MFAZO algorithm optimally balances distinct objectives during weight optimization by mimicking the social and natural foraging behaviors of American zebras in a way that adjusts the exploration and exploitation stages dynamically. In the feature extraction process employing the MW-RBM, the MFAZO considers the complex objectives such as maximizing the feature relevance and reducing the overfitting. It achieves this by estimating the candidate weight solutions over a fitness function that includes distinct criteria, enabling the algorithm to prioritize the solutions that offer the best trade-offs rather than tuning a single measure. The “magnified feeding” strategy improves the search efforts around the promising areas, improving the local exploitation without prematurely converging, while the randomization maintains the diversity to discover new regions in a solution space. This careful balancing guarantees that the optimized weights not only enhance the discriminative power of the extracted features but also maintain the mode efficiency and generalizability, resulting in more robust and accurate attack identification in the IoT healthcare data. The pictorial representation of the designed MW-RBM-based feature extraction is displayed in Fig. 3.

## Multistacked deep learning technique and encryption method for handling the healthcare data

### Residual GRU

ResGRU^36^ is developed by adopting the residual block in the GRU model to extract the time series feature from the given samples. The GRU model is used to manage the long-term memory and the sequential problems in the network structure. The hidden units in the network are replaced using the gate structure to evaluate the significant data and avoid unnecessary data. The input representations are determined using the reset gate${s_t}$ with the support of the update gate${y_t}$ and previous memory. The more state information is achieved based on the larger value of${y_t}$. The number of data is determined using the reset gate in the node ${g_t}$. Further, the hidden layer is calculated in the present movement using the update gate. The calculation process is evaluated in the following Eqs. (13) to (16).

13 $${y_t}=\sigma ({X_y}{z_t}+{V_y}{g_{t - 1}}+{c_z})$$

14 $${s_t}=\sigma ({X_s}{z_t}+{V_s}{g_{t - 1}}+{c_s})$$

15 $${g_t}=\tanh ({X_g}{z_t}+{V_g}({s_t} \otimes {g_{t - 1}})+{c_g})$$

16 $${g_t}=(1 - {y_t}) \otimes {g_{t - 1}}+{y_t} \otimes {g_t}$$

In this, the weight matrices are indicated as ${X_y}$,${X_s}$,${X_g}$,${V_y}$,${V_s}$,${V_g}$. The bias value is indicated as ${c_y}$,${c_s}$,${c_g}$. The current movement hidden layer output is indicated as${g_t}$, and the Hadamard product is denoted as $\otimes$.

The degradation performance has been solved using the residual network. The residuals $F(z)=G(z) - z$are learned by the network, increasing the learning feature of the network. The identity mapping is applied by the accumulation layer when the residual is considered zero. The sum of the result of the GRU’s last layer is equal to the output of the residual block. The residual block is evaluated using Eq. (17).

17 $${z_R}=relu(B{n_{\gamma ,\beta }}(z)+h({y_t}))$$

Here, the ReLU activation and the batch normalization function are specified as $relu(.)$and $BN(.)$. The learnable variables are indicated as $\gamma$and $\beta$. The correlation between the time sequence data and the information is remembered using the ResGRU. It supports to improve the performance of detection.

### Novel MSRes-GRU for monitoring system

This work developed the MSRes-GRU model for effective attack detection in IoT nodes. The proposed approach is designed by the integration of the multiscale stacked layer into the ResGRU network to perform the detection process.

**Actual threat models:** The suggested approach targets a range of crucial threat approaches normally encountered in IoT-based healthcare systems. Ransomware attacks pose an important concern by encryption patient data and requiring payment, highly disrupting crucial healthcare services. The technique’s real-time attack identification concentrates on recognising such malicious encryption behavior early, eliminating widespread data lockout. Man-in-the-middle (MITM) attacks, where the adversaries alter and intercept communication among IoT servers and systems, are resolved through the utilization of blockchain’s secure data hashing and immutable ledger, guaranteeing data integrity and eliminating unauthorized tampering. In addition, the approach considers the adversarial machine learning attacks, where the attackers craft the deceptive inputs to fool the detection algorithms; the multiscale residual GRU’s effective temporal feature learning improves the resilience over the sophisticated evasion strategies by capturing the tiny anomalies across multiple time scales. Thus, these defenses offer a detailed security posture against both emerging and conventional cyber threats in the healthcare IoT environments.

**Difference of MSRes-GRU from conventional ResGRU or GRU-CNN hybrids: **The MSRes-GRU differs from the traditional residual GRU and GRU-CNN hybrids highly in its architectural design and its capability to capture the temporal patterns at distinct scales. Unlike the standard residual GRU approaches that employ the residual connections to a single sequence scale, the MSRes-GRU utilizes the stacked layers that execute the input data at distinct temporal scales or resolutions simultaneously, enabling it to retrieve both coarse and fine-grained features from IoT data. This multiscale mechanism improves the sensitivity of the model to distinct temporal dynamics, which is important for identifying the evolving and complex attack patterns. Contrasted to the GRU-CNN hybrids, which integrate the convolutional layers with GRUs, the designed MSRes-GRU solely concentrates on the temporal design with residual pathways to resolve the vanishing gradient problems, thus enhancing the learning stability and depth without the added complexity of the CNN layers. This makes the designed MSRes-GRU highly specialized for the time-series attack identification operations in IoT systems, providing better precision and robustness in recognizing the subtle changes over dynamic time patterns.

Here, the resultant feature $Ad_{k}^{{fe}}$ from the MW-RBM is provided as input to the MSRes-GRU approach. In general, the detection task requires multiple convolution mechanisms to evaluate the different-sized feature maps to perform effective detection. Hence, the multiscale stacked convolution is incorporated to the ResGRU to improve the performance of attack detection. The MW-RBM features are given to the multiscale stacked convolution layer to evaluate the different low-level features from the given input at the same time. With the help of the convolutional kernel, the effective features are extracted from the input character to get the superior feature output. By using the multiscale stacked convolution, the different scale representational features are retrieved. These retrieved features are determined utilizing the ResGRU to perform the attack detection. The two gates in the GRU, such as the update gate and the reset gate, help to evaluate how much data to keep and how much to avoid. The residual connection in the GRU allows the model to propagate data in an easier manner using the deeper network. The vanishing gradient issue can be addressed by the residual connection. The combinational network ResGRU can able to manage long-term dependencies effectively. This network easily allows the gradient flow based on the shortcut connection to preserve the information over a long time. The suggested MSRes-GR network detects the attack from the given MW-RBM features.

**Multi-scale feature extraction in security improvement:** Multiscale feature extraction improves the security by allowing the system to validate the data at distinct granularities and temporal resolutions, enabling it to identify both long-term attack patterns and short-term anomalies that may be missed by single-scale approaches. In the IoT-based healthcare context, where threats can change in timing and complexity, this multiscale mechanism enables the approach, most importantly the multiscale stacked residual GRU to capture the subtle changes in the data flow, device behavior and the communication patterns. As an outcome, it becomes more effective in recognizing sophisticated attacks such as time-distributed malware activity. This layered and deeper understanding of the input data increases the capability of the technique to preemptively identify and respond to threats, thus increasing the overall security and robustness of the system.

**Blockchain transactions by GRU outputs:** The blockchain transactions are triggered by the results of the MSRes-GRU approach on the basis of the detection results of the IoT node activities. When the GPU executes the optimized features and recognizes suspicious or normal behavior, its outcome serves as a decision signal: legitimate data packs or flagged attack alerts. For each estimated data instance, where the normal health data or identified anomaly, the system produces a specific transaction that is securely hashed, recorded and time-stamped on a blockchain. This guarantees a transparent and immutable ledger of entire IoT security events and communications. By connecting the GRU outcomes directly to blockchain transactions, the model ensures that each identified event is logged in real-time, allowing auditability, traceability and tamper-proof protection of the healthcare data in its entire lifecycle. 

**Fig. 4:**
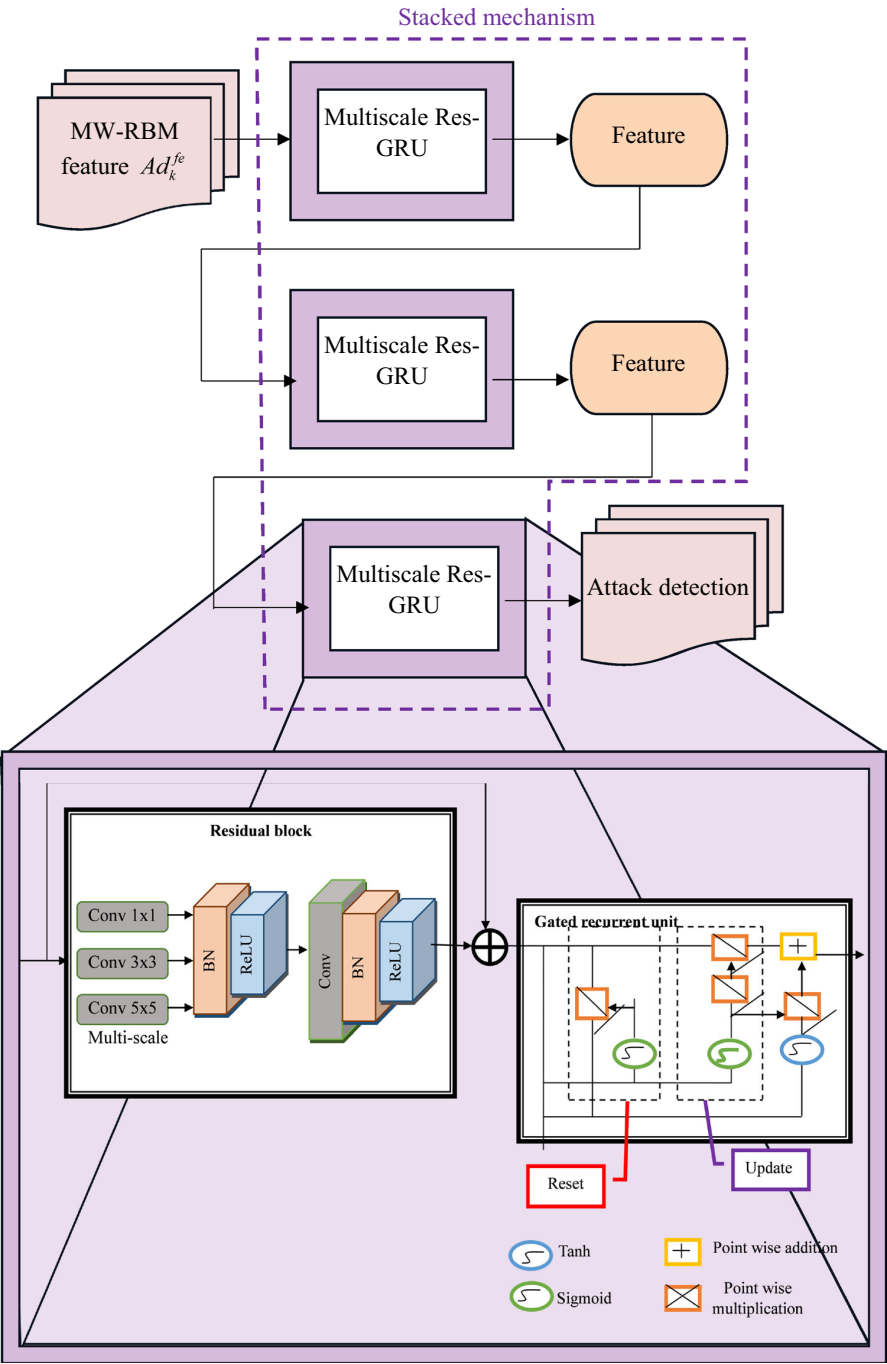
Illustration of MSRes-GRU for Attack detection.

**Use of MSRes-GRU compared to otheradvanced temporal sequence modeling approaches:** The use of MSRes-GRU is justified over other advanced temporal sequence modeling models because of its unique capability to capture intricate temporal dependencies across distinct time scales while addressing common complexities such as vanishing gradients. Unlike the conventional LSTMs or GRUs that perform on an individual temporal scale, the multiscale model executes the input sequences at dynamic resolutions, enabling the approach to identify both long-term patterns and short-term anomalies crucial for recognizing sophisticated IoT healthcare threats. The incorporation of the residual connections then improves the learning stability and enables deeper network models without affecting the performance, which is mostly a problem in other hybrid or recurrent approaches. Compared to hybrid techniques such as GRU-CNNs, which include spatial feature extraction but increase the computational complexity, the designed MSRes-GRU concentrates purely on the temporal dynamics, making it highly effective and well-suited for time series attack identification, where the temporal context is important. Thus, the designed MSRes-GRU provides a powerful and balanced model that integrates multi-scale validation and gradient flow optimization, leading to robustness and superior accuracy in identifying the temporal anomalies in the healthcare IoT data. The structural view of the implemented MSRes-GRU model for attack detection in the IoT node is shown in Figure 4.

### Data encryption using HPE

To secure the IoT-enabled healthcare system, the medical data needs an effective encryption model. Hence, this work develops the HPE to perform the encryption and decryption process to preserve the healthcare details from third parties.

In general, the blockchain network is used to store sensitive healthcare data to preserve it. Here, the HPE is constructed to perform the encryption and decryption task to improve the security of the medical data Db_w_. The HE is a complex mathematical process that is performed to encrypt the data to preserve the relationship in both sets of elements. In addition, the polynomial multiplication is added to the HE for secure communication, and the construction is represented as HPE. For secure encryption, the private and the public keys are produced as the security key. The four phases in the HPE are encryption key generation, encrypting the healthcare data into ciphertext, decrypting using the private key and determining transmitted data.

The polynomial function $f(k)$is assigned with the coefficient degree $K$. The regression coefficient and the average standard deviation are computed in the encryption without performing the decryption. There are $n$ degrees of polynomials in the ring $rn={K_q}(y)/f(y)$. The polynomial $f(y)$selected for the operation ${y^n}+1$. The general addition and the multiplication of the polynomial is compoundwise coefficient addition. The polynomial function$f(y)={y^n}+1$ is used to perform the key generation, where the $n$ has power of two. The uniform random element in the secret key is defined by the ring element. In addition, an error term is added to the key. The random element is added in the public key with the error term for the encryption. The authorized person is provided with the private key and the public key is available for the device platform for encryption protocol. The encryption is performed by the degree of the polynomial. Here, the encryption time relies on the degree of the polynomial. The public key is added with the appropriate error value to generate the public key.

**Fig. 5:**
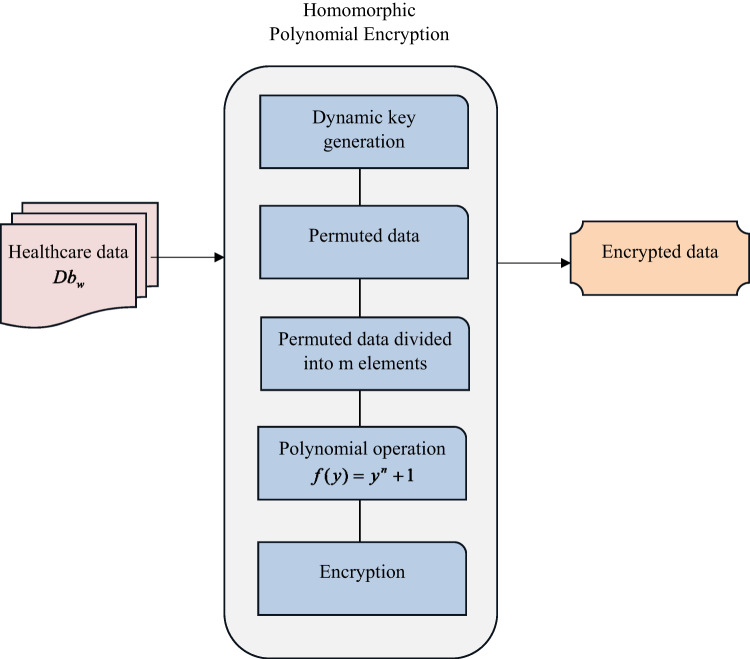
Diagrammatic view of the proposed HPE-based data encryption.

The ideal principle of the polynomial mechanism helps in generating a particular encryption. Based on this mechanism, the given healthcare data are encrypted. This encryption provides enhanced security and solves the complexity of the underlying process. The diagrammatic view of HPE-based encryption is shown in Fig. 5.

**Contributions of HPE and MSRes-GRU in threat detection and mitigation:** The HPE and MSRes-GRU play important roles in identifying and mitigating threats in IoT-based healthcare devices. The HPE guarantees that the sensitive patient data remains encrypted in the entire processing and transmission, allowing computations on the encrypted data without revealing it to powerful attackers. This maintains the privacy and secures against data breaches or interception attempts, such as those viewed in MITM attacks or ransomware targeting original data. At the same time, the MSRes-GRU performs well in validating the temporal patterns in the encrypted, optimized features retrieved from the IoT device data. Its multiscale model captures both long-term trends and short-term changes, making it relatively effective at recognizing the subtle signs of adversarial manipulations or temporal patterns that existing approaches may miss. By identifying the anomalies early, the designed MSRes-GRU triggers the alerts that eliminate the ransomware spread and other threats. Hence, the HPE secures the data environment, whereas the MSRes-GRU offers robust threat identification, developing a resilient model that protects the healthcare data availability and integrity against evolving cyber attacks.

## Results and discussion

### Experimental setup

The suggested IoT-aided secure healthcare system was evaluated using the Python platform, and the results were obtained. The comparative algorithms used for the performance analysis were Gorilla Troops Optimizer (GTO)^37^, Border Collie Optimization (BCO)^38^, Fossa Optimization Algorithm (FOA)^39^ and AZOA^34^. Other classifiers such as DNN^40^, LSTM^41^, SVM^42^ and GRU^36^ and existing cryptography algorithms such as Data Encryption Standard (DES)^43^, Advanced Encryption Standard (AES)^44^, Elliptic Curve Cryptography (ECC)^45^ and Rivest-Shamir-Adleman (RSA)^46^ were employed. The implementation details of blockchain are given in Table 2.

**Table 2:** Implementation details of blockchain.

Attribute	Description
Blockchain Type	Permissioned blockchain restricted to authorized healthcare participants
Consensus Mechanism	Lightweight trust-based consensus (similar to PBFT/PoA) for low-latency IoT transactions
Smart Contracts	Used for authentication, automated access control, and transaction validation
Data Hash Storage	On-chain: Patient data hashes & timestamps; Off-chain: Complete medical records
Security Attributes	Immutability, transparency, confidentiality, auditability, and resistance to tampering
Scalability Support	Optimized storage with off-chain data handling and efficient consensus

### Performance analysis

Accuracy, Balanced Accuracy (BA), bookmaker and F1-score are determined using Eqs. (18) to (21).

18 $$Acy=\frac{{{B^p}+{B^n}}}{{{B^p}+{B^n}+{Y^p}+{Y^n}}}$$

19 $$BA=\frac{{{B^p}+{B^n}}}{2}$$

20 $$BM=\operatorname{Re} +Spy - 1$$

21 $$F1S=2*\frac{{{B^p}*{B^n}}}{{{B^p}+{B^n}}}$$

‘False Discovery Rate (FDR), Fowlkes–Mallows (FM), False Negative Rate (FNR) and False Omission Rate (FOR)’ are evaluated using Eqs. (22) to (25).

22 $$FDR=\frac{{{Y^p}}}{{{Y^p}+{Y^p}}}$$

23 $$FM=\sqrt {\Pr s \times \operatorname{Re} }$$

24 $$Fnr=\frac{{{Y^n}}}{{{Y^n}+{Y^p}}}$$

25 $$FOR=\,\frac{{{Y^n}}}{{{Y^n}+{B^n}}}$$

‘False Positive Rate (FPR), Negative Predictive Value (NPV) and precision’ value are demonstrated using Eqs. (26) to (28).

26 $$FPR=\frac{{{Y^p}}}{{{Y^p}+{B^n}}}$$

27 $$Npv=\frac{{{B^n}}}{{{B^n}+{Y^n}}}$$

28 $$\Pr s=\frac{{{B^p}}}{{{B^p}+{Y^p}}}$$

Prevalence Threshold (PT), sensitivity, specificity and markedness computed using Eqs. (29) to (32).

29 $$PT=\,\frac{{\sqrt {{B^p} \times {Y^p}} - {Y^p}}}{{{B^p} - {Y^p}}}$$

30 $$Sty=\frac{{{B^p}}}{{{B^p}+{Y^n}}}$$

31 $$Spy=\frac{{{B^p}}}{{{B^n}+{Y^p}}}$$

32 $$MK=\Pr s+NPV - 1$$

The terms ${B^p}$and ${B^n}$represent the ‘true positive and true negative’. ${Y^p}$and ${Y^n}$denote the ‘false positive and false negative’.

### Convergence validation of the suggested MFAZO algorithm

**Fig. 6:**
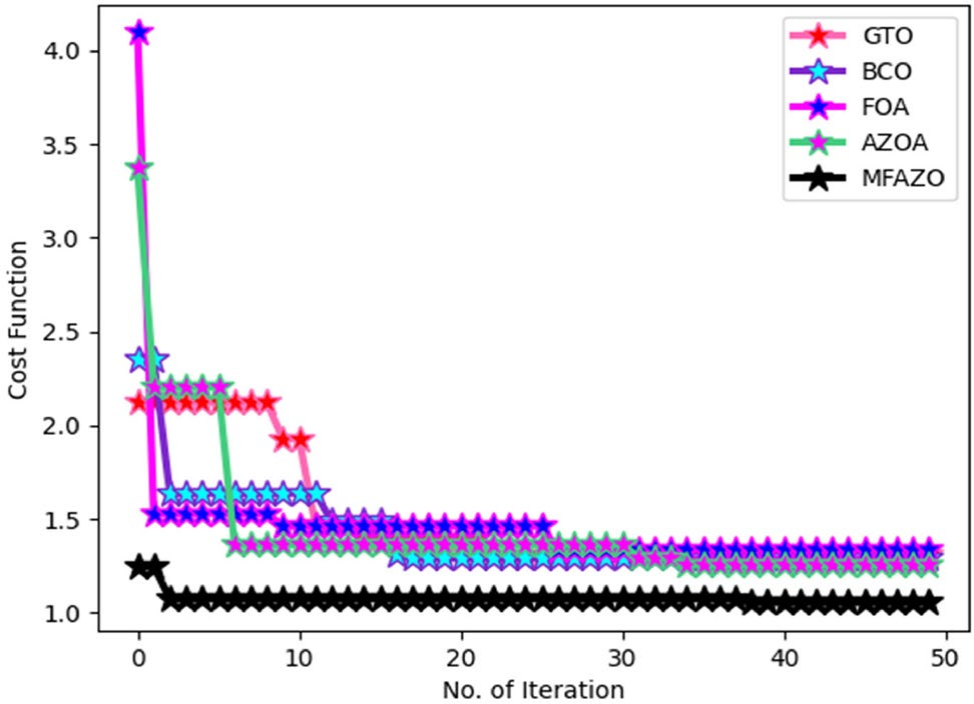
Cost function validation of the implemented MFAZO algorithm over existing algorithms.

The cost function estimation of the designed MFAZO algorithm over other standard algorithms for optimal feature selection is shown in Fig. 6. The designed algorithm handles the weight optimization in the feature extraction process to achieve the optimal result. The functionality of the designed algorithm is evaluated using the determination of the convergence by varying the iteration values. The convergence of the implemented MFAZO is enhanced by 13% of GTO, 8% of BCO, 5% of FOA, and 4% of AZOA. The obtained result of MFAZO highlighted the maximum convergence over other comparative algorithms, which showed that MFAZO achieved the optimal solution to the problem.

### Statistical calculation of the presented MFAZO algorithm

The evaluation of the implemented MFAZO algorithm is determined using statistical analysis. Here, the proposed MFAZO is analyzed over other traditional algorithms shown in Table 3. Here, the designed MFAZO performed the optimal feature selection in the feature extraction process. The performance and the characteristics of the proposed optimization strategy are determined using this analysis. From the table analysis, the MFAZO algorithm achieved better values than 24.2% of GTO, 22.4% of BCO, 27% of FOA, and 19% of AZOA. The achieved statistical result ensured a better value obtained from the MFAZO algorithm.

**Table 3:** Statistical analysis of the implemented MFAZO over classical Algorithms.

Terms	GTO [29]	BCO [30]	FOA [31]	AZOA [26]	MFAZO
Best	1.310157	1.290821	1.340032	1.254909	1.054526
Worst	2.121316	2.350717	4.094548	3.370103	1.247091
Mean	1.505295	1.419547	1.467286	1.44876	1.076706
Median	1.310157	1.290821	1.461727	1.363083	1.074634
Std	0.314069	0.236547	0.38277	0.38459	0.035811

### ROC calculation of MSRes-GRU model for detection

**Fig. 7:**
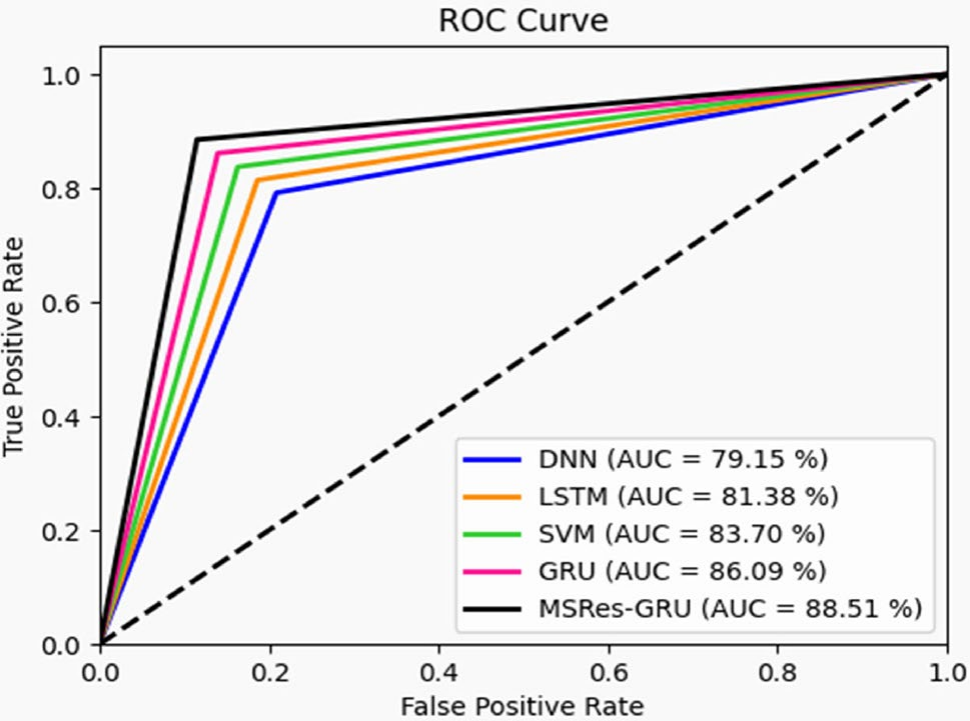
ROC analysis of the designed MSRes-GRU for the attack detection over existing techniques.

The suggested detection framework, called MSRes-GRU, is validated with other traditional classifiers in terms of ROC analysis, as shown in Fig. 7. Here, the ‘true positive rate and the false positive rate’ are used to determine the ROC value. This analysis helps to evaluate how well the model performs the detection task without any false assumptions. While taking the FPR as 0.2, the designed MSRes-GRU attained a higher value than 11.09% of DNN, 11.03% of LSTM, 8.06% of SVM, and 5.5% of GRU, respectively. From the outcome, the designed approach attained a better detection rate than other comparative methods.

### Epoch-based comparative analysis of the implemented attack detection model

**Fig. 8:**
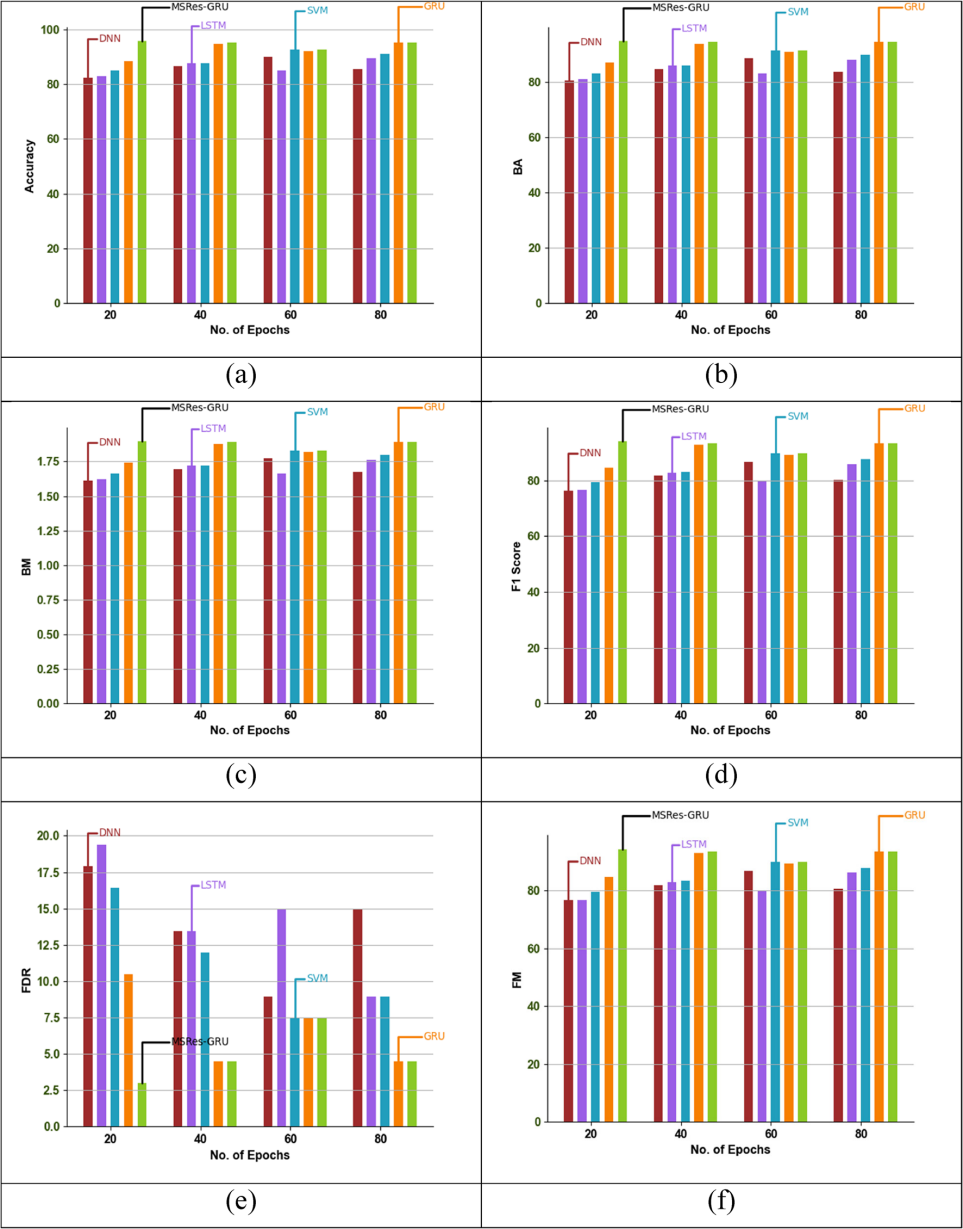

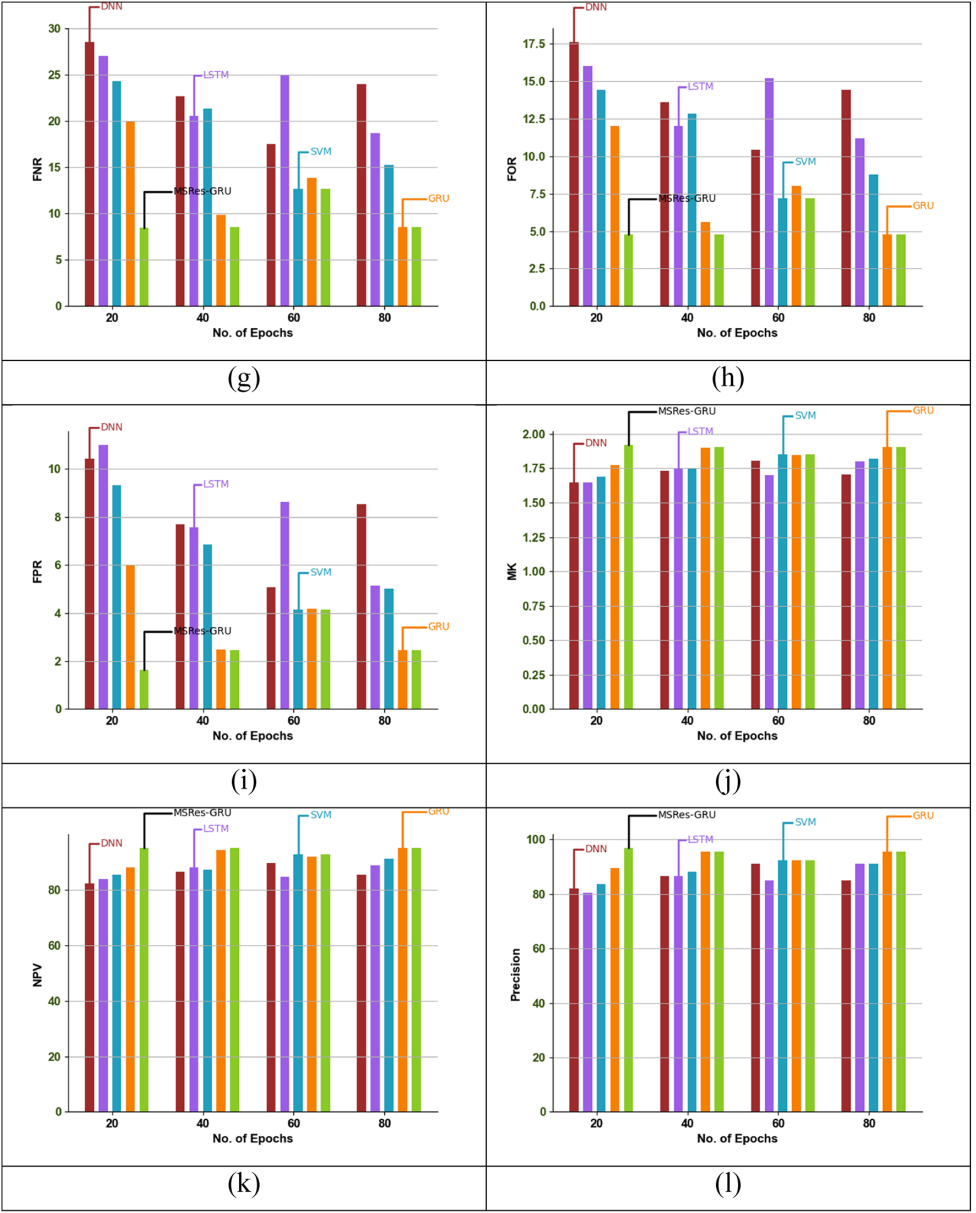

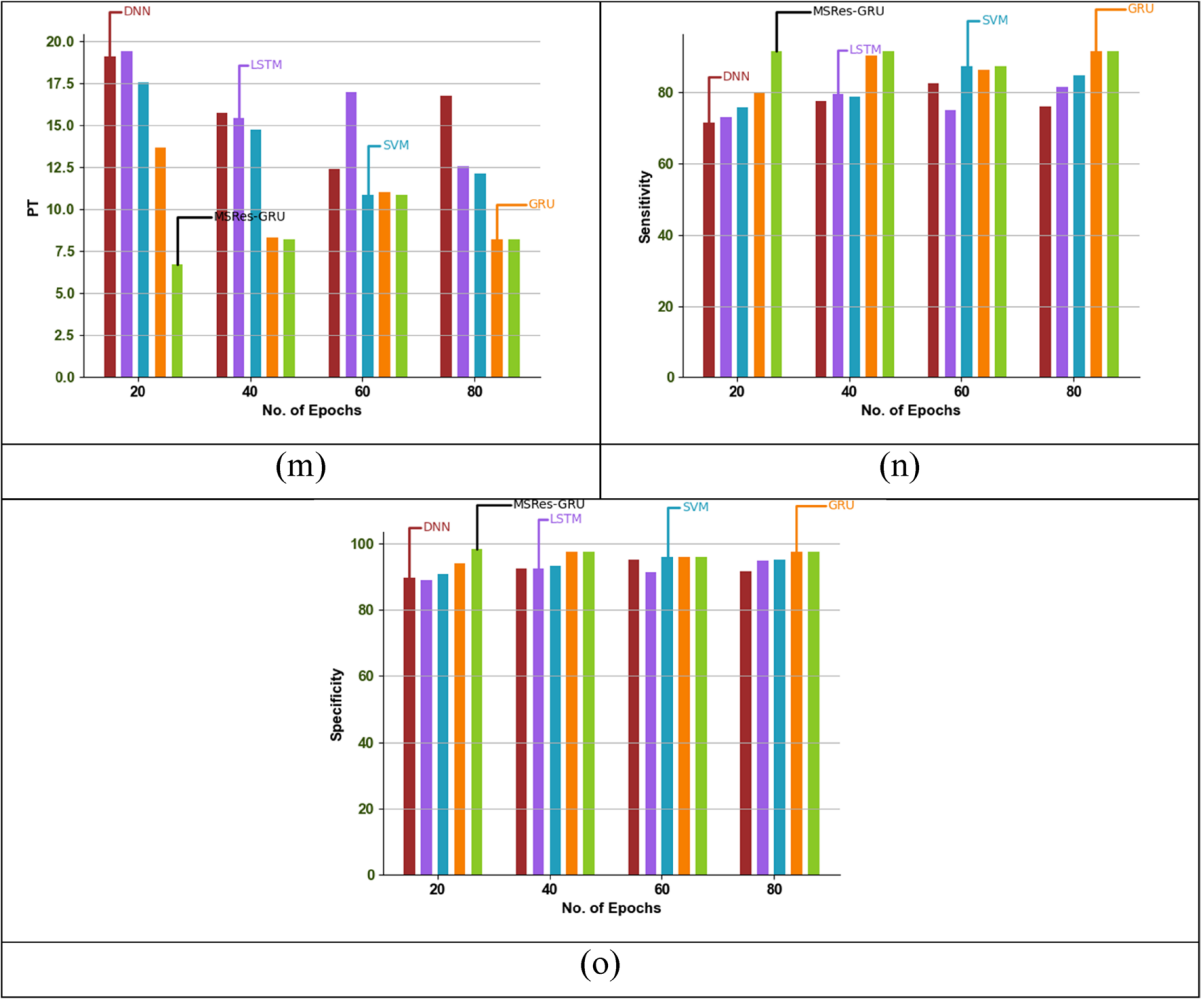
Epoch-based validation of the designed detection approach MSRes-GRU compared with other techniques regarding “(a) Accuracy, (b) BA, (c) BM, (d) F1score, (e) FDR, (f) FM, (g) FNR, (h) FOR, (i), FPR, (j) MK, (k) NPV, (l) Precision, (m) PT, (n) Sensitivity, and (o) Specificity”.

Epoch-based analysis of the MSRes-GRU model compared with other attack detection such as GRU and SVM, is demonstrated in Fig. 8. For the given dataset, the attack detection is analysed based on varying the epoch function within the range of 20 to 80. Based on the graphical analysis, the recommended MSRes-GRU attained higher results than 16.2% of DNN, 14.7% of LSTM, 12.6% of SVM, and 7.3% of GRU when taking the epoch function as 20. From the comparative results, the MSRes-GRU model achieved a better value than any other models.

### K-fold-based performance analysis of the designed MSRes-GRU model

K-fold-based performance analysis of the designed attack detection model over other detection models is shown in Table 4. Here, different measures are used to evaluate the performance of the approach, such as accuracy, specificity, FPR, MCC, etc. From the validation, the implemented detection approach MSRes-GRU attained higher accuracy than 8.6% of DNN, 8.6% of LSTM, 11.9% of SVM, and 3.2% of GRU, respectively. Based on the highlighted results and the remaining results from the table, the detection model MSRes-GRU achieved better results than other methods.

**Table 4:** K-fold-based analysis of the designed MSRes-GRU for detection compared with other Models.

Kfold	DNN [32]	LSTM [33]	SVM [34]	GRU [28]	MSRes-GRU
Accuracy					
1	85.94868	85.97353	90.81942	87.49862	90.83079
2	86.75607	85.17497	92.41369	92.89981	92.89981
3	86.46779	85.48522	86.63043	86.74266	91.94185
4	80.9072	87.14261	82.40712	88.90684	92.11076
5	85.65184	80.71502	89.99949	94.14141	96.22934
Specificity					
1	99.26463	99.26586	99.5422	99.35467	99.5435
2	99.31354	99.21529	99.62886	99.65404	99.65404
3	99.29358	99.23457	99.30502	99.30907	99.60285
4	98.93726	99.33226	99.03945	99.43559	99.61163
5	99.24396	98.92881	99.49581	99.71811	99.82213
FPR					
1	7.353673	7.341418	4.578013	6.453317	4.565028
2	6.864584	7.847051	3.711433	3.459607	3.459607
3	7.064242	7.65434	6.949818	6.90932	3.971483
4	10.62743	6.677392	9.605466	5.644146	3.883746
5	7.560367	10.71189	5.04187	2.818936	1.778707
NPV					
1	85.94669	85.97179	90.8198	87.4987	90.83055
2	86.75372	85.17476	92.41312	92.89959	92.89959
3	86.46786	85.48483	86.62899	86.74356	91.94217
4	80.90843	87.14399	82.40667	88.90726	92.11129
5	85.65215	80.71237	90.00099	94.14124	96.22941
MCC					
1	3.889185	3.893674	4.994589	4.1972	4.998323
2	4.04702	3.743922	5.470036	5.630551	5.630551
3	3.987491	3.800836	4.02108	4.04119	5.320811
4	3.052937	4.12184	3.279835	4.50949	5.3729
5	3.83109	3.028836	4.775839	6.084173	7.03484
CSI					
1	21.01617	21.04933	30.07339	23.33072	30.10527
2	22.17777	19.98792	34.62804	36.2613	36.2613
3	21.74117	20.38799	21.9864	22.14341	33.15686
4	15.55343	22.75468	16.92154	25.8393	33.6679
5	20.60497	15.40511	28.11642	41.13069	52.59697
BM					
1	1.210258	1.210613	1.305595	1.2349	1.305918
2	1.222649	1.199234	1.352682	1.369481	1.369481
3	1.218007	1.203531	1.220615	1.222295	1.337531
4	1.150844	1.228796	1.165948	1.261365	1.342809
5	1.20586	1.149203	1.285224	1.419285	1.535264

### Comparative validation of the security framework

**Fig. 9:**
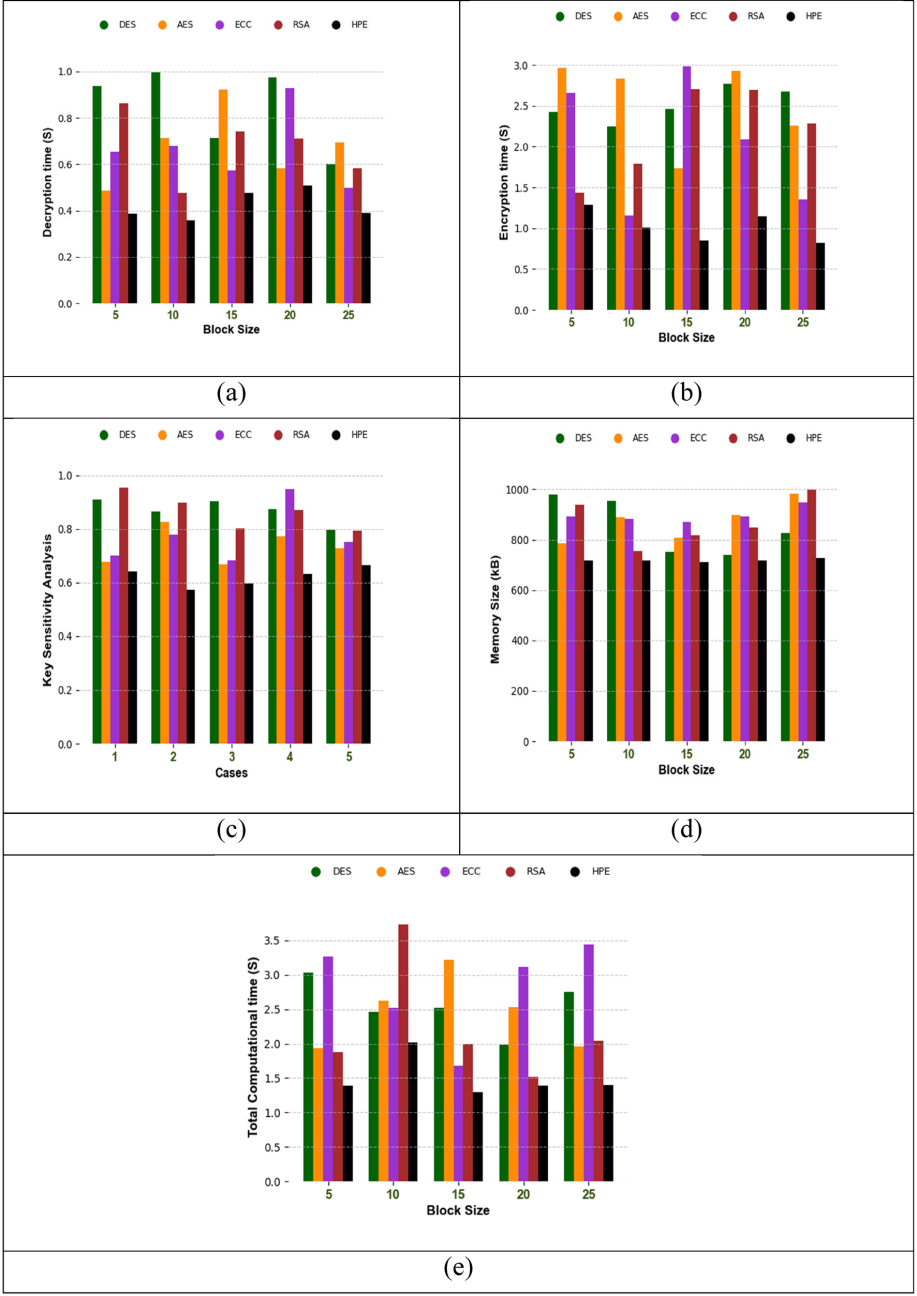
Comparison of the suggested HPE-based security model over other classical frameworks in terms of “(a) Decryption time, (b) Encryption time, (c) Key sensitivity, (d) Memory size, and (e) Total computational time”.

The comparative analysis of the recommended security model, HPE, analyzed over other standard techniques, as expressed in Fig. 9. One of the major concerns in the encryption-based security model is the time taken to encrypt or decrypt the data. In addition, the memory size and the key sensitivity analysis are mainly determined to determine the performance of the security model. From the graphical images, the designed HPE achieved a fewer decryption time than 12.5% of DES, 2.5% of AES, 6.2% of ECC and 10.7% of RSA. From the result, the recommended HPE security model has better encryption performance by minimizing the encryption, decryption and computational time.

### Computational time and interference time analysis

Tables 5 and 6 show the computational time and inference time validations of the suggested approach over existing algorithms and techniques. The computational time is the overall time and resources required to fulfil the task, including the overall aspects of the system’s operation, while the inference time is a particular component of that, referring only to the time a trained approach takes to execute the new data and generate an outcome, excluding the training time. This analysis shows that the designed approach takes 13 s for computational time and 12.1 s for the inference time, which are lower than the previous models. Thus, the outcomes ensured that the recommended model achieves much lower computational and inference times than the existing models and obtains better efficiency.

**Table 5:** Computational time analysis.

Terms	Computational time (seconds)
Based on algorithms	
GTO [29]	15.8861
BCO [30]	16.3154
FOA [31]	18.9985
AZOA [26]	15.8470
MFAZO	13.009599
Based on models	
DNN [32]	14.5962
LSTM [33]	16.3123
SVM [34]	18.1289
GRU [28]	14.3358
MSRes-GRU	13.009599

**Table 6:** Inference time analysis.

Terms	Inference time (seconds)
Based on algorithms	
GTO [29]	16.5941
BCO [30]	15.1968
FOA [31]	17.2984
AZOA [26]	14.1985
MFAZO	12.19685
Based on models	
DNN [32]	15.9698
LSTM [33]	14.8684
SVM [34]	17.9841
GRU [28]	13.1975
MSRes-GRU	12.19685

### Computational complexity analysis

The computational complexity validation of the recommended MFAZO is shown in Table 7 over conventional algorithms. It analyzes the algorithm’s resource usage as the input size grows. It concentrates on how effectively the algorithms scale and categorizes the computational problems. Here, the variables${M_{itr}},{P_{non}},{C_{len}}$ define the maximum iteration, number of population and chromosome length. From the experimental result, the designed MFAZO shows higher computational efficiency than the conventional algorithms.

**Table 7:** Computational complexity analysis.

Algorithms	Computational complexity
GTO [29]	$O\left[ {{M_{itr}}+1+{P_{non}}+1+{C_{len}}} \right]$
BCO [30]	$O\left[ {{M_{itr}}+{P_{non}}+2+{C_{len}}} \right]$
FOA [31]	$O\left[ {{M_{itr}}+1+{P_{non}}+2+{C_{len}}+3} \right]$
AZOA [26]	$O\left[ {{M_{itr}}+1+{P_{non}}+{C_{len}}+2} \right]$
MFAZO	$O\left[ {{M_{itr}}+{P_{non}}+{C_{len}}} \right]$

### Security analysis

Table 8 presents the security analysis of the designed approach over conventional approaches. In this analysis, the attack detection rate, FPR, and FNR are discussed. From the analysis, it has been highlighted that the designed MSRes-GRU model attains 88.5% attack detection, 5.5% false positive and 6.8% false negative rates. Hence, it is confirmed that the designed MSRes-GRU model provides more security than the conventional approaches.

**Table 8:** Security analysis.

Models	Attack detection rate	FPR	FNR
DNN [32]	79.1	10.5	12.3
LSTM [33]	81.4	9.8	11
SVM [34]	83.7	8.6	10.1
GRU [28]	86	7.9	8.9
MSRes-GRU	88.5	5.5	6.8

### Efficiency analysis

**Fig. 10:**
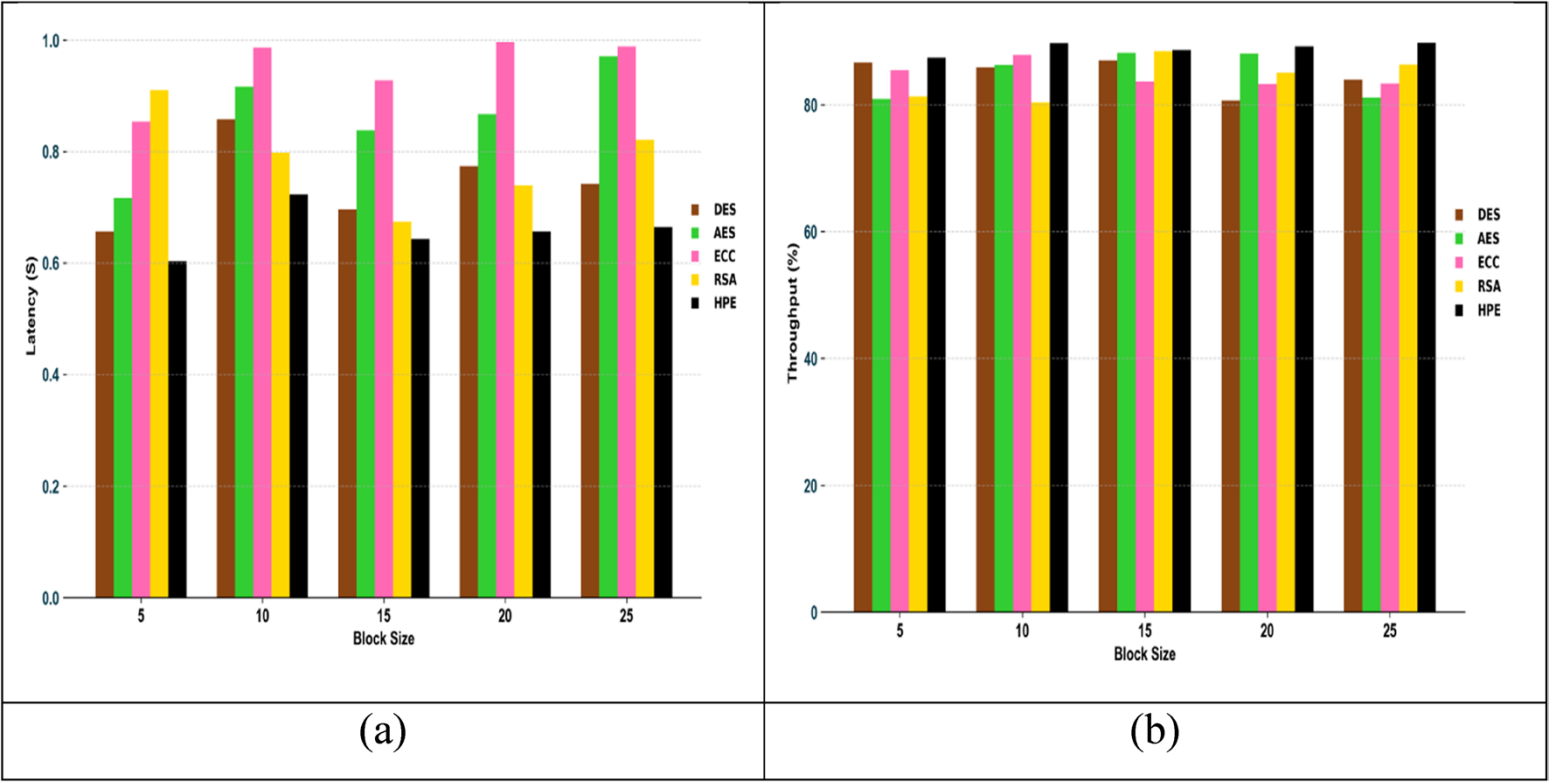
Efficiency analysis of the implemented model in terms of “(a) Latency and (b) Throughput”.

Figure 10 displays the efficiency analysis of the designed framework. Here, the latency and throughput of the suggested approach are analyzed. For the distinct block size values, the effectiveness is validated in this validation. When considering the 15th block size value, the suggested HPE’s throughput is 3.48%, 2.32%, 6.97%, and 1.74% enhanced than the conventional models such as DES, AES, ECC and RSA. Therefore, it is confirmed that the recommended HPE is more effective than the conventional methods.

### Optimization impact analysis

**Fig. 11:**
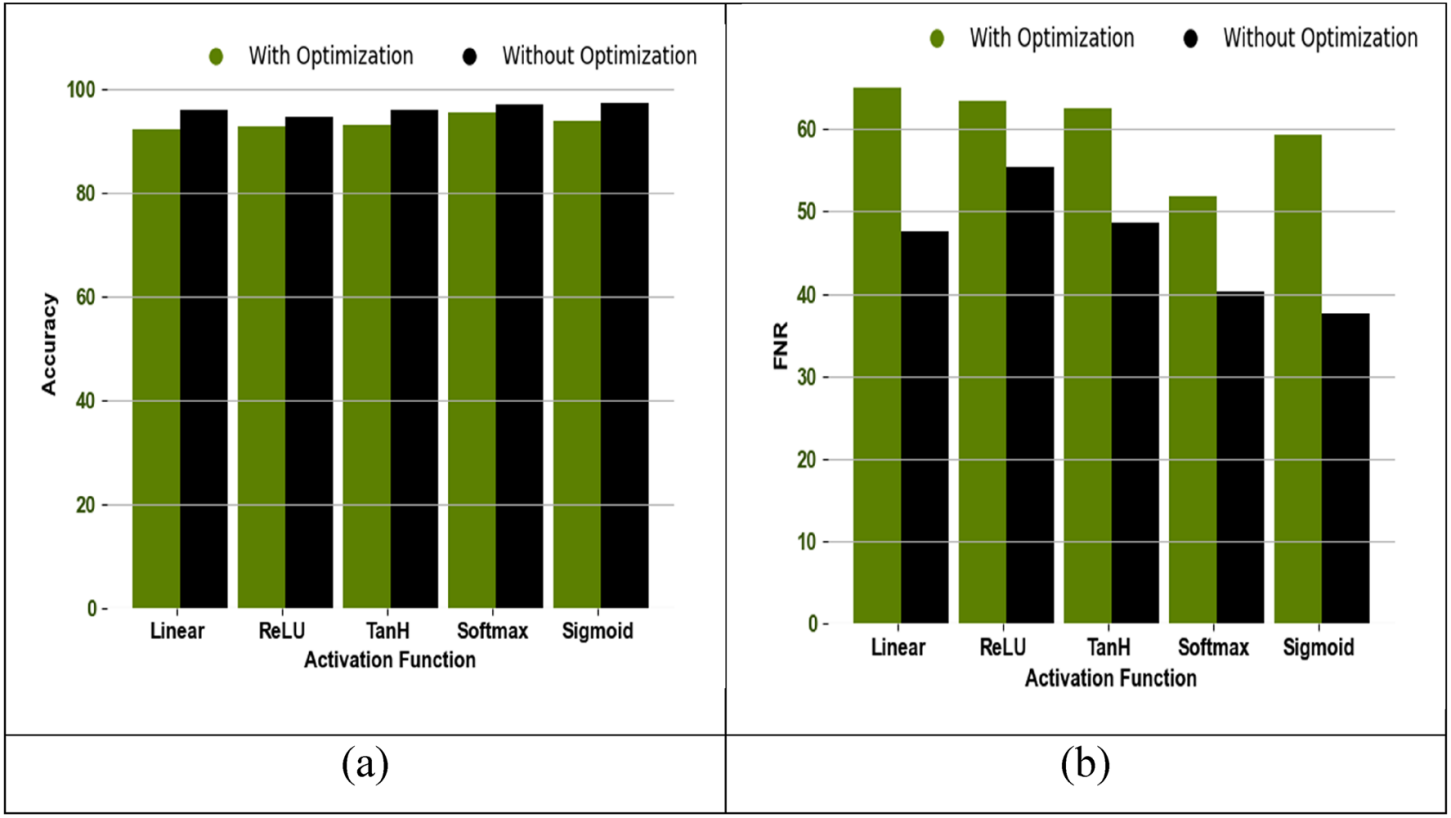
Analysis of optimization impact in terms of “(a) Accuracy, and (b) FNR”.

Figure 11 analyzes the optimization impact of the recommended work. The designed model utilizes the MFAZO for the weight optimization, and its impact in the suggested work is estimated in terms of accuracy and FNR. From this experiment, it is showcased that the recommended work achieves high accuracy and low FNR rates when involving the optimization process.

### Accuracy Analysis

Table 9 shows the accuracy analysis of the suggested work by employing some standard datasets such as UNSW-NB15, CICIDS and MIMIC-III. These are the publicly available datasets for research in different domains: healthcare and cybersecurity. Therefore, the suggested model is investigated with these standard datasets. The accuracy of the designed MSRes-GRU is 93.15%, 92.83% and 91.9% when validating with the UNSW-NB15, CICIDS and MIMIC-III datasets, respectively, for the first k-fold value. These values are higher than the conventional models for three datasets. Hence, it is portrayed that the designed UNSW-NB15, CICIDS and MIMIC-III show high accuracy and robustness.

**Table 9:** Accuracy Analysis.

Kfold	DNN [32]	LSTM [33]	SVM [34]	GRU [28]	MSRes-GRU
UNSW-NB15					
1	88.256	84.672	88.512	90.816	93.152
2	83.744	87.04	84.864	90.368	92.288
3	85.28	85.984	87.552	91.36	94.752
4	78.368	83.328	90.784	90.784	95.424
5	82.112	90.048	83.008	88.96	94.272
CICIDS					
1	87.392	84.704	82.944	91.52	92.832
2	78.848	83.072	83.488	92.672	96.224
3	77.952	79.968	90.24	90.24	92
4	76.16	90.336	92.288	90.016	95.232
5	81.888	87.744	87.712	90.816	95.968
MIMIC-III					
1	87.584	84.48	83.264	86.336	91.904
2	77.504	80.16	85.76	91.488	94.464
3	86.688	85.28	86.08	90.496	91.84
4	76.864	84.64	85.248	90.848	90.848
5	82.944	80.576	90.848	87.136	95.2

### Scalability analysis

**Fig. 12:**
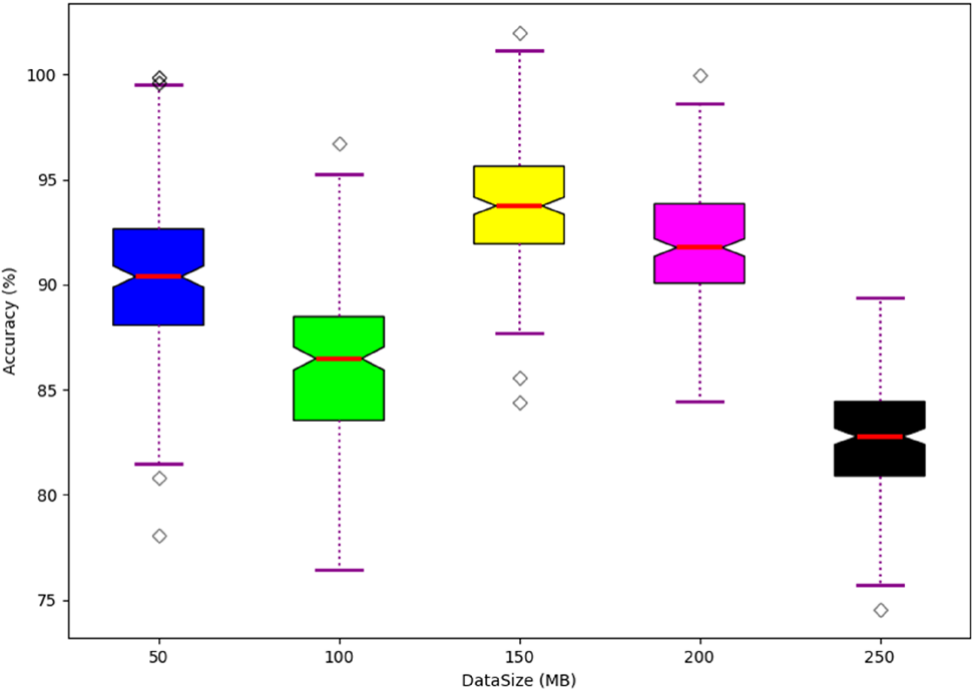
Scalability Analysis.

The scalability analysis is shown in Fig. 12. It is the operation of testing and validating the capability of the system to handle the enhanced workloads, such as more users or transactions, while maintaining acceptable functionality. This analysis utilizes the data size for the validation and estimates the accuracy of the overall process. From the validation, it is confirmed that the designed model achieves high scalability even when the transaction or the number of users is higher.

## Conclusion

In this research, a novel framework has designed for the healthcare system to ensure secure data transmission and enhance the ability of data protection. For IoT-enabled healthcare management, the blockchain-based cryptography mechanism was implemented for securing sensitive medical data. At first, the required attack detection data and the medical data were taken from the standard data resources. Optimal feature extraction is carried out using the proposed MW-RBM along with weight optimization using MFAZO for the attack detection data. The resultant optimal features were used to perform the attack detection, where the MSRes-GRU model was developed. In the end, healthcare data were secured using the implemented HPE-based encryption framework. This HPE ensures the security of the medical data. The functionality of the framework was evaluated by the comparative analysis. From the analysis, the accuracy of the MSRes-GRU-aided detection was enhanced by 8.1% of DNN, 16.7% of LSTM, 4.3% of SVM, and 3.7% of GRU, respectively. From the obtained results, the designed methodology provides a robust result than other comparative algorithms.

**Further directions:** Further directions for this research include exploring large-scale and diverse healthcare IoT data sources that capture a large range of real-world attack scenarios, such as emerging attacks like Advanced Persistent Threats (APTs) and zero-day exploits. In addition, examining the deployment models that help federated learning and edge computing could improve real-time threat identification while minimizing latency and preserving patient privacy. On the blockchain front, employing hybrid blockchain models, integrating private and public chains may provide enhanced scalability, quick consensus approaches and fine-grained access control associated with sensitive healthcare environments. In addition, combining cross-chain interoperability could allow secure data sharing across distinct healthcare platforms and providers. These directions concentrate on bolstering the approach’s efficiency, applicability and robustness in highly distributed and complex IoT healthcare ecosystems.

## Data Availability

The data underlying in the article are taken from the following link: Attack Detection dataset: The corresponding dataset taken from the link “https://www.kaggle.com/datasets/venkatakanumuru/kddcup99csv” Access date: 2025-02-14. Healthcare dataset: The diabetes dataset utilized in this work is taken from the link “https://www.kaggle.com/datasets/akshaydattatraykhare/diabetes-dataset” Access date: 2025-02-06.
